# LINC00665 promotes breast cancer progression through regulation of the miR-379-5p/LIN28B axis

**DOI:** 10.1038/s41419-019-2213-x

**Published:** 2020-01-06

**Authors:** Wei Ji, Yu-Ling Diao, Yi-Ran Qiu, Jie Ge, Xu-Chen Cao, Yue Yu

**Affiliations:** 1 Public Laboratory, Tianjin Medical University Cancer Institute and Hospital, National Clinical Research Center for Cancer, 300060 Tianjin, China; 2 Key Laboratory of Cancer Prevention and Therapy, 300060 Tianjin, China; 3 Tianjin’s Clinical Research Center for Cancer, 300060 Tianjin, China; 4 Key Laboratory of Breast Cancer Prevention and Therapy, Tianjin Medical University, Ministry of Education, 300060 Tianjin, China; 5 The First Department of Breast Cancer, Tianjin Medical University Cancer Institute and Hospital, National Clinical Research Center for Cancer, 300060 Tianjin, China

**Keywords:** Long non-coding RNAs, Breast cancer

## Abstract

Breast cancer is the most common malignant tumor among women worldwide. Although increasing evidence indicates that long noncoding RNAs (lncRNAs) play critical roles during breast tumorigenesis and progression, the involvement of most lncRNAs in breast cancer remains largely unknown. In the current study, we demonstrated that LINC00665 promotes breast cancer cell proliferation, migration, and invasion. Accumulating evidence indicates that many lncRNAs can function as endogenous miRNA sponges by competitively binding common miRNAs. In this study, we demonstrated that LINC00665 functions as a sponge for miR-379-5p, reducing the ability of miR-379-5p to repress LIN28B. LINC00665 promoted breast cancer progression and induced an epithelial–mesenchymal transition-like phenotype via the upregulation of LIN28B expression. Clinically, LINC00665 expression was increased but miR-379-5p expression was decreased in breast cancer tissues compared with that in normal breast tissues in the TCGA database. Furthermore, the expression of LINC00665 was negatively related with miR-379-5p expression. Collectively, our results reveal the LINC00665–miR-379-5p–LIN28B axis and shed light on breast cancer therapy.

## Introduction

Breast cancer is one of the most common malignant tumor and the main cause of cancer-associated mortality in women worldwide^1^. Although earlier diagnosis and systemic therapy have improved the prognosis of breast cancer patients, recurrence, metastasis and drug resistance are barriers to the successful treatment of patients with breast cancer. Moreover, our understanding of the pathogenesis and mechanisms of breast cancer remains greatly limited. Thus, identifying new genes and pathways involved in breast cancer will aid the development of faster and safer diagnostic methods and improve breast cancer prognosis and treatment.

Over 90% of human genes can be transcribed into RNAs, but only 1–2% can encode proteins^2^. Long noncoding RNAs (lncRNAs) are a class of non-coding RNAs longer than 200 bp. Approximately 50,000 lncRNAs have been discovered, but only a few lncRNAs have undergone preliminary study^3^. LncRNAs are highly conserved, and although they do not encode proteins themselves, they regulate target genes by affecting transcription, epigenetics, and posttranslational modifications^4^. Recent accumulating evidence supports the involvement of lncRNAs in regulation of chromatin remodeling, transcription, posttranscription, and translation^5–8^.

LncRNAs are frequently dysregulated in multiple malignancies and act as either tumor suppressors or oncogenes and as important regulators during tumorigenesis and cancer progression; moreover, they are helpful diagnostic and prognostic markers^9,10^. LINC00665 is located at chromosome 19q13.12. Several studies have demonstrated that LINC00665 functions as an oncogene in tumorigenesis and progression. Recently, microarray analysis revealed LINC00665 as being upregulated in lung adenocarcinoma^11^. Database analysis also revealed that LINC00665 is overexpressed in hepatocellular carcinoma and might contribute to cancer progression by regulating cell cycle pathways^12^. As stated above, the expression of LINC00665 is increased in lung adenocarcinoma, and LINC00665 upregulation is associated with poor outcome in patients with lung adenocarcinoma. Moreover, Linc00665 promotes lung cancer progression by acting as a miRNA sponge for miR-98 to facilitate AKR1B10 expression via ERK signaling^13^. In addition, downregulation of LINC00665 reduced resistance to gefitinib through interaction with EZH2 and inactivation of the PI3K/AKT pathway^14^. However, knowledge about the role of LINC00665 in breast cancer is still limited.

In the current study, we investigated the role of LINC00665 in breast cancer development and progression. We demonstrated that LINC00665 promotes cancer progression and induces an epithelial–mesenchymal transition (EMT)-like phenotype in breast cancer by sponging miR-379-5p. Furthermore, we identified LIN28B as a direct target of miR-379-5p. Together, our study reveals that the LINC00665–miR-379-5p–LIN28B axis in breast cancer and provide a novel mechanism explaining breast cancer progression.

## Results

### Depletion of LINC00665 suppresses breast cancer progression

**Fig. 1:**
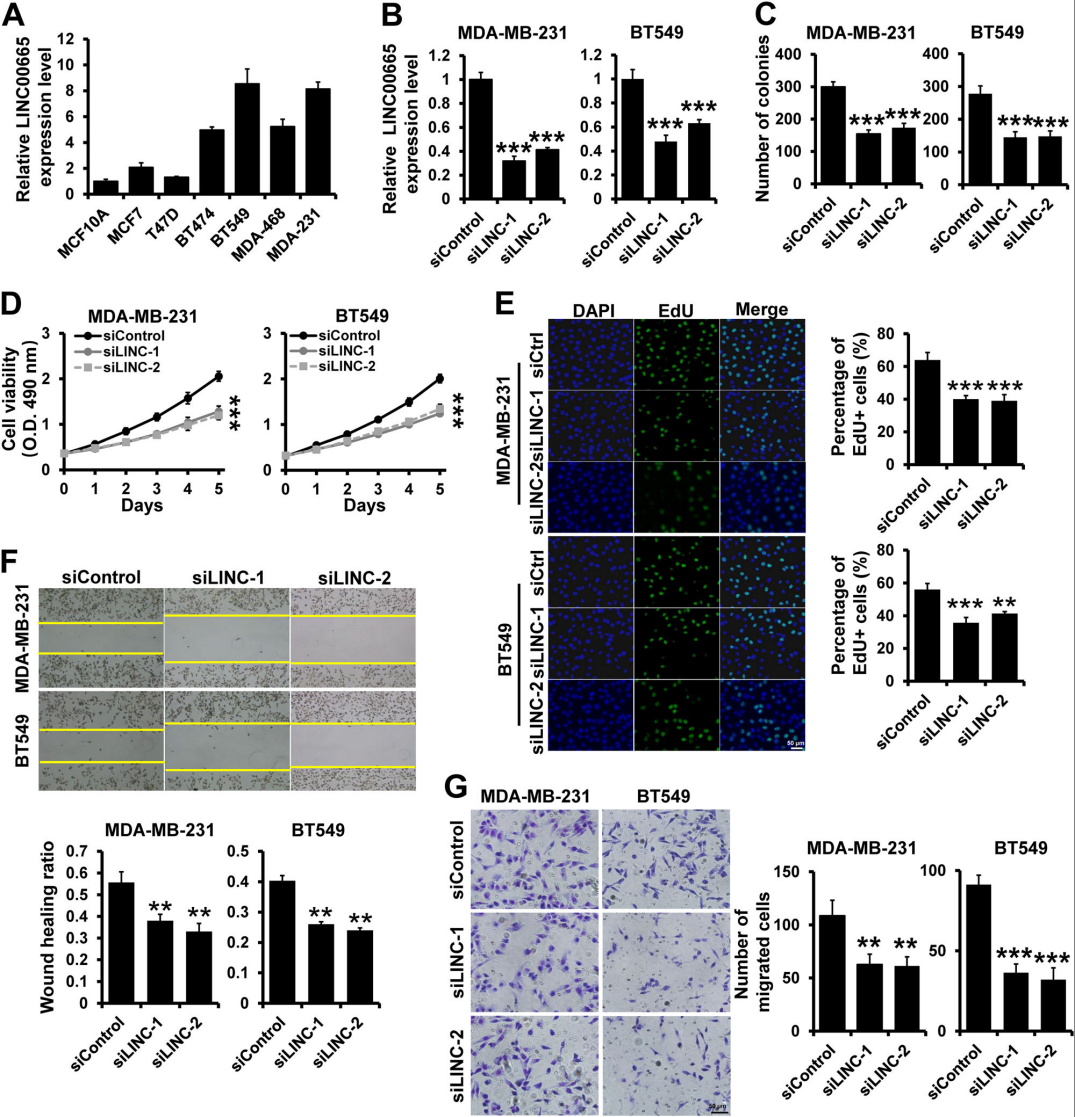
Depletion of LINC00665 inhibits breast cancer cell proliferation, migration, and invasion. **a** LINC00665 expression in normal breast cell lines and breast cancer cell lines was determined by RT-qPCR. **b** LINC00665 expression in MDA-MB-231 and BT549 cells transfected with siRNAs targeting LINC00665 was determined by RT-qPCR. **c**–**e** Cell growth inhibition was evaluated by colony formation **c**, MTT **d**, and EdU **e** assays in the cells described in **b**. **f** Representative images of wound-healing assays after scratch wounding of the cells described in **b**. **g** Transwell invasion assay of the cells described in **b**. ***P < 0.001.

To demonstrate the role of LINC00665 in breast cancer development and progression, we determined the expression of LINC00665 in six breast cancer cell lines and the normal breast epithelial cell line MCF10A by reverse transcription quantitative polymerase chain reaction (RT-qPCR). We observed that the expression of LINC00665 was upregulated in most of the breast cancer cell lines compared to that in MCF10A cells. In addition, LINC00665 was highly expressed in TNBC cell lines compared to that in ER+ breast cancer cell lines (Fig. 1a). Consistent with the results from cell lines, the expression of LIC00665 is increased in patients with TNBC from TCGA database (Fig. S1). We further explored the effect of LINC00665 on breast cancer proliferation, migration, and invasion in vitro by introducing LINC00665 siRNAs into the MDA-MB-231 and BT549 cell lines, which have higher endogenous LINC00665 expression levels than the other breast cancer cell lines (Fig. 1b). The results of MTT, colony formation, and EdU assays indicated that depletion of LINC00665 suppressed breast cancer cell proliferation (Fig. 1c, e). Furthermore, the results of Transwell and wound-healing assays indicated that LINC00665 depletion inhibited the migration and invasive abilities of MDA-MB-231 and BT549 cells (Fig. 1f, g). Next, we generated stable LINC00665-depleted MDA-MB-231 cells (shLINC00665) as well as control cell line (shControl) (Fig. S2A). 231-shControl or 231-shLINC00665 cells were inoculated into female SCID mice and tumor growth was monitored. We observed that the tumor volume was significantly decreased in shLINC00665 group compared with this in control group (Fig. S2B and C). Together, these results indicate that depletion of LINC00665 inhibits breast cancer progression.

### Overexpression of LINC00665 promotes breast cancer progression

**Fig. 2:**
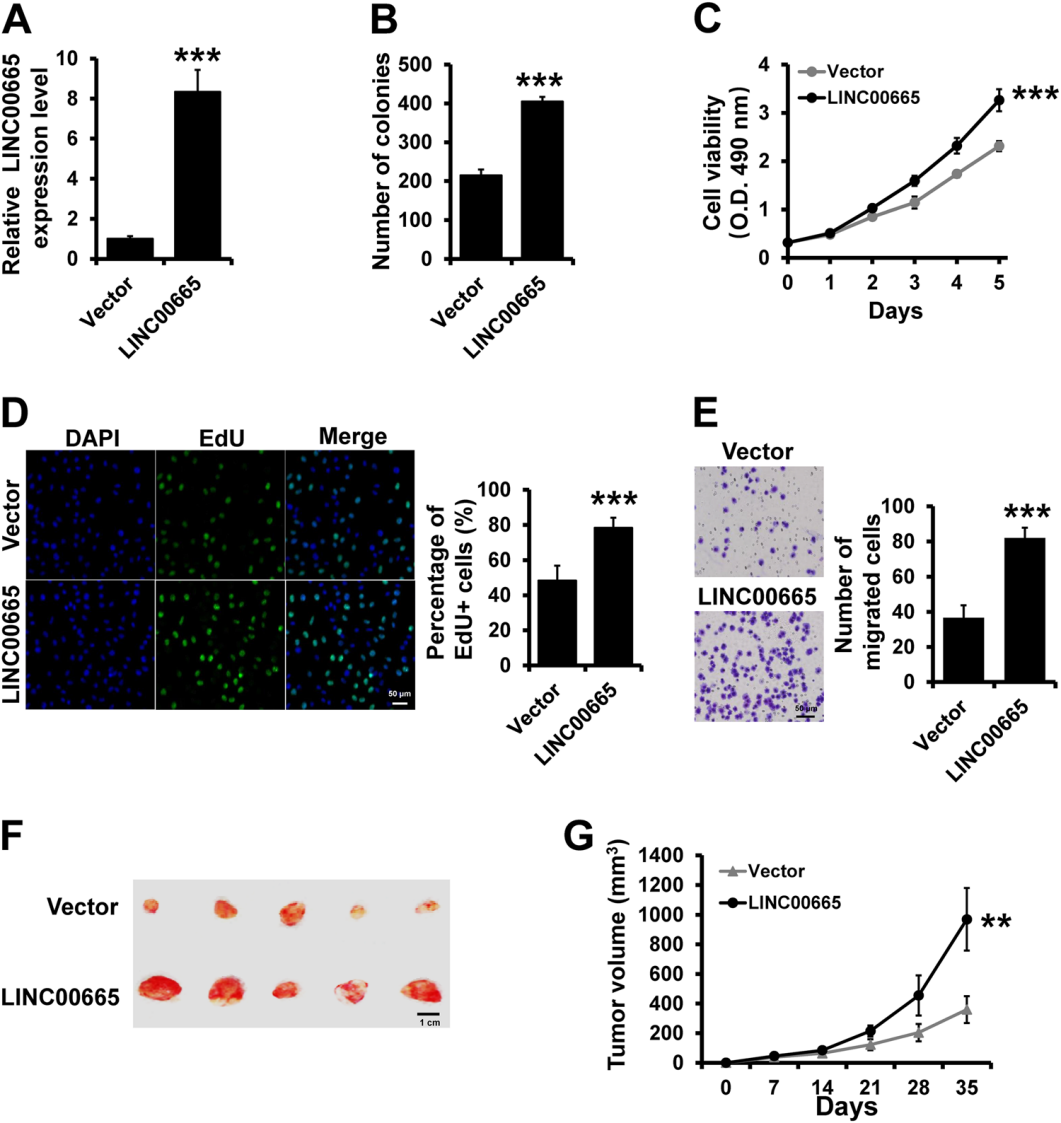
Overexpression of LINC00665 promotes breast cancer progression. **a** LINC00665 expression in T47D cells transfected with the LINC00665 expression vector or empty vector was determined by RT-qPCR. **b**–**d** Growth inhibition was evaluated by colony formation **b**, MTT **c**, and EdU **d** assays in the cells described in **a**. **e** Transwell invasion assay of the cells described in **a**. **f** Representative photographs of the tumors formed by T47D-LINC00665 or control cells at the time of harvest. **g** Volume of tumors in xenograft mice injected with T47D-LINC00665 or control cells at the indicated times. ***P < 0.001, **P < 0.01.

We next investigated whether LINC00665 overexpression can influence cancer progression in breast cancer cells. We generated stable LINC00665-overexpressing T47D cells by transfecting the pcDNA3.1-LINC00665 expression plasmid as well as a vector control cell line (Fig. 2a). The results of MTT, colony formation, and EdU assays indicated that overexpression of LINC00665 promoted the proliferation of T47D cells (Fig. 2b–d). The Transwell assay results showed that overexpression of LINC00665 promoted the invasion of T47D cells (Fig. 2e). Next, T47D-LINC00665 or T47D-vector cells were inoculated into female SCID mice and tumor growth was monitored. We observed that the tumor volume was significantly increased in LINC00665 group compared with that in control group (Fig. 2f, g). Together, these results indicate that LINC00665 promotes breast cancer progression.

### LINC00665 induces an EMT-like phenotype in breast cancer cells

**Fig. 3:**
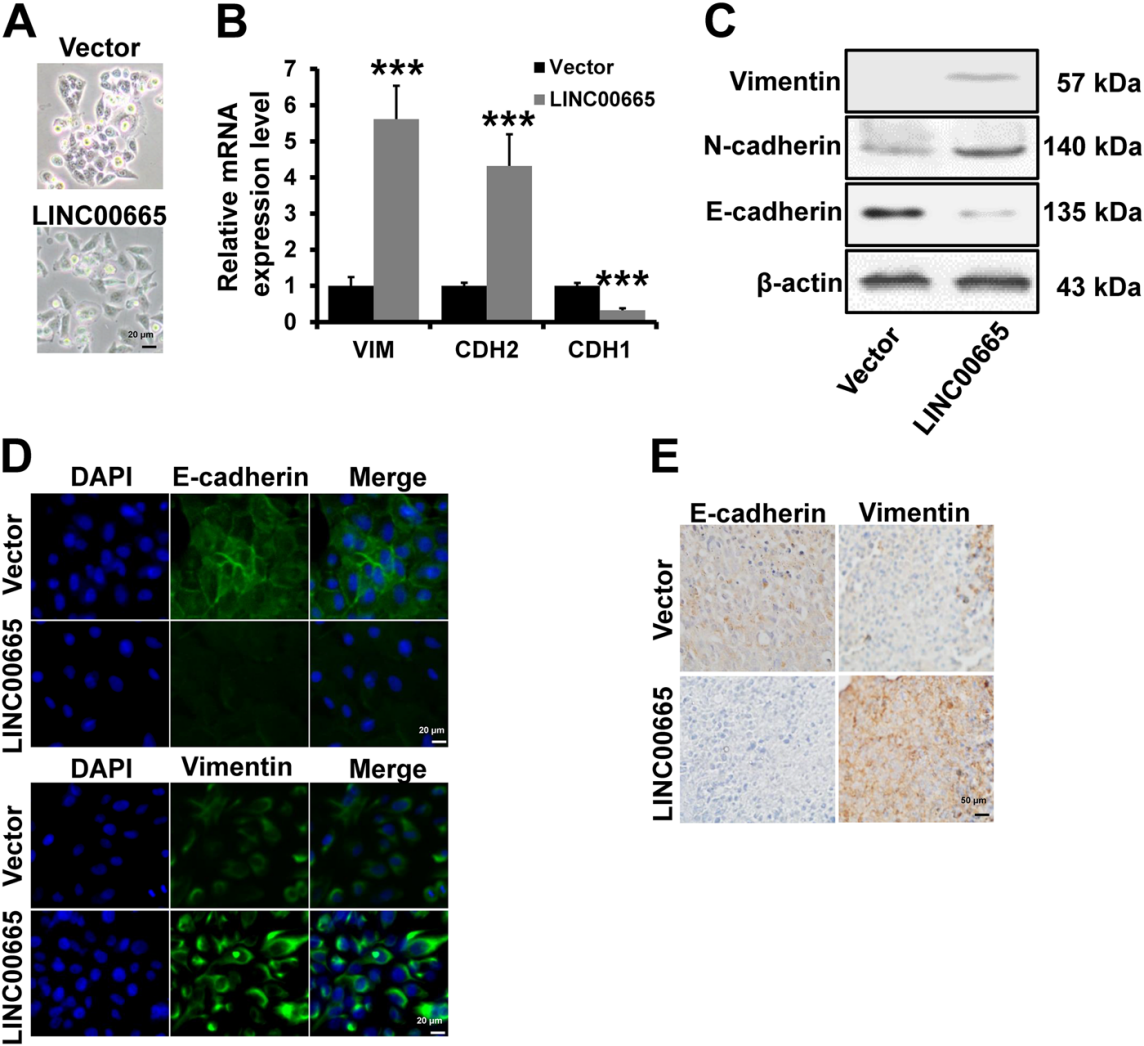
LINC00665 induces an EMT-like phenotype. **a** Images of LINC00665-overexpressing T47D cell and control cell morphology. **b** and **c** mRNA **b** and protein **c** expression levels of EMT markers in the cells described in **a** were evaluated by RT-qPCR and western blotting, respectively. **d** Immunofluorescence analyses of EMT markers in the cells described in **a**. **e** The expression of EMT markers in T47D-LINC00665 and T47D-vector xenograft tumors was examined by immunohistochemical staining. ***P < 0.001.

EMT is one of the critical mechanisms involved in breast cancer progression in which epithelial cells acquire an invasive mesenchymal phenotype. We observed that T47D-vector cells retained their cobblestone-like morphology, whereas LINC00665-overexpressing cells displayed a fibroblast-like morphology (Fig. 3a). We examined the expression of mesenchymal and epithelial markers by RT-qPCR (Fig. 3b), western blotting (Fig. 3c) and immunofluorescence (Fig. 3d). LINC00665-overexpressing T47D cells exhibited significant downregulation of E-cadherin but dramatic upregulation of the mesenchymal markers Vimentin and N-cadherin. Immunohistochemical staining confirmed the upregulation of Vimentin and downregulation of E-cadherin in tumors from T47D-LINC00665 mice relative to the expression of these proteins in tumors from T47D-vector mice (Fig. 3e). Together, these results suggest that LINC00665 induces an EMT-like phenotype in breast cancer cells.

### LINC00665 regulates miR-379-5p by acting as a ceRNA

**Fig. 4:**
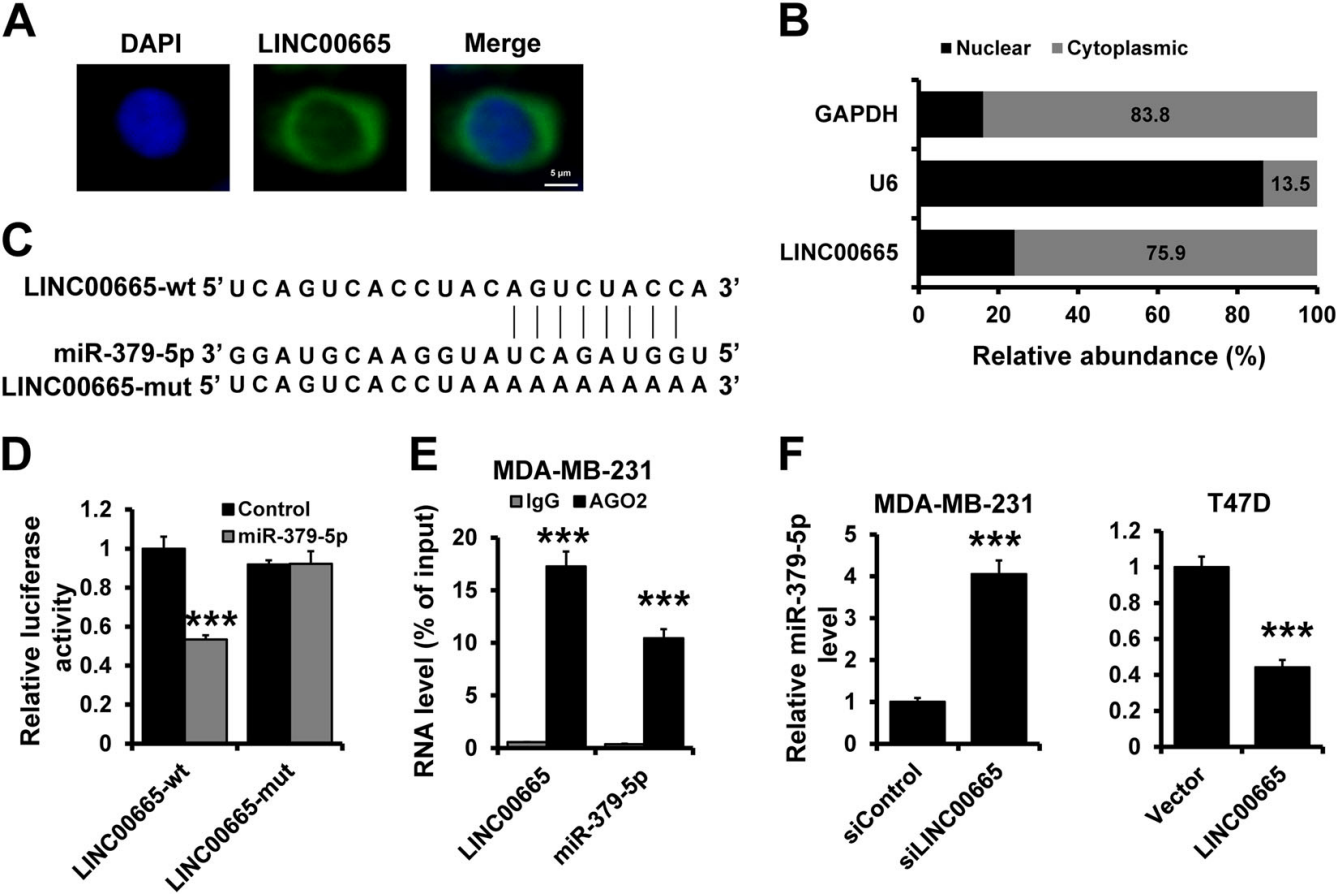
LINC00665 acts as a sponge for miR-379-5p. **a** Representative FISH images indicating the subcellular localization of LINC00665 in MDA-MB-231 cells (green). Nuclei were stained with DAPI (blue). **b** Relative LINC00665 expression levels in the nuclear and cytoplasmic fractions of MDA-MB-231 cells. **c** The predicted binding site of miR-379-5p in LINC00665. The mut sequence contains an 8-base mutation in the miR-379-5p target seed region. **d** A dual luciferase reporter assay was performed to validate the interaction between miR-379-5p and LINC00665. **e** RIP analysis of endogenous AGO2 binding to RNA in MDA-MB-231 cells. IgG was used as the control. The LINC00665 and miR-379-5p levels were determined by RT-qPCR. **f** The miR-379-5p expression levels in LINC00665-depleted MDA-MB-231 cells (left) and LINC00665-overexpressing T47D cells (right), as well as in the corresponding control cells, were determined by RT-qPCR. ***P < 0.001.

To further investigate the potential mechanisms underlying the oncogenic role of LINC00665, we determined the subcellular localization of LINC00665 by using fluorescence in situ hybridization (FISH) (Fig. 4a) and subcellular fractionation followed by RT-qPCR (Fig. 4b). We observed that LINC00665 was mainly expressed in the cytoplasm. Cytoplasmic lncRNAs can function as miRNA sponges by competitively binding common miRNAs. LINC00665 was predicted to function as a sponge for miR-379-5p (Fig. 4c). To further evaluate whether miR-379-5p could bind to LINC00665, we constructed luciferase reporters containing wild-type (LINC00665-wt) LINC00665 and LINC00665 with mutation of the miR-379-5p-binding site (LINC00665-mut). As shown in Fig. 4d, cotransfection of LINC00665-wt but not LINC00665-mut with the miR-379-5p mimic significantly decreased luciferase activity in 293FT cells. In addition, LINC00665 and miR-379-5p were significantly enriched in AGO-containing microribonucleoprotein complexes by RNA immunoprecipitation (RIP) assay, suggesting that both LINC00665 and miR-379-5p bind directly to AGO2 in breast cancer cells (Fig. 4e). Moreover, the expression of miR-379-5p was significantly up-regulated in LINC00665-depleted MDA-MB-231 cells (Fig. 4f; left) but was decreased in T47D-LINC00665 cells compared to that in control cells, as evidenced by RT-qPCR (Fig. 4f; right). Next, we generated stable LINC00665-overexpressing MDA-MB-231 cells, LINC00665/miR-379-5p-overexpressing MDA-MB-231 cells, as well as control cell line (Fig. S3A). We observed that overexpression of miR-379-5p eliminates LINC0065-induced breast cancer progression both in vitro and in vivo (Fig. S3B–3G). Taken together, our results indicate that LINC00665 promotes breast cancer progression in a manner partly dependent on miR-379-5p sponging.

### LIN28B is a target of miR-379-5p

**Fig. 5:**
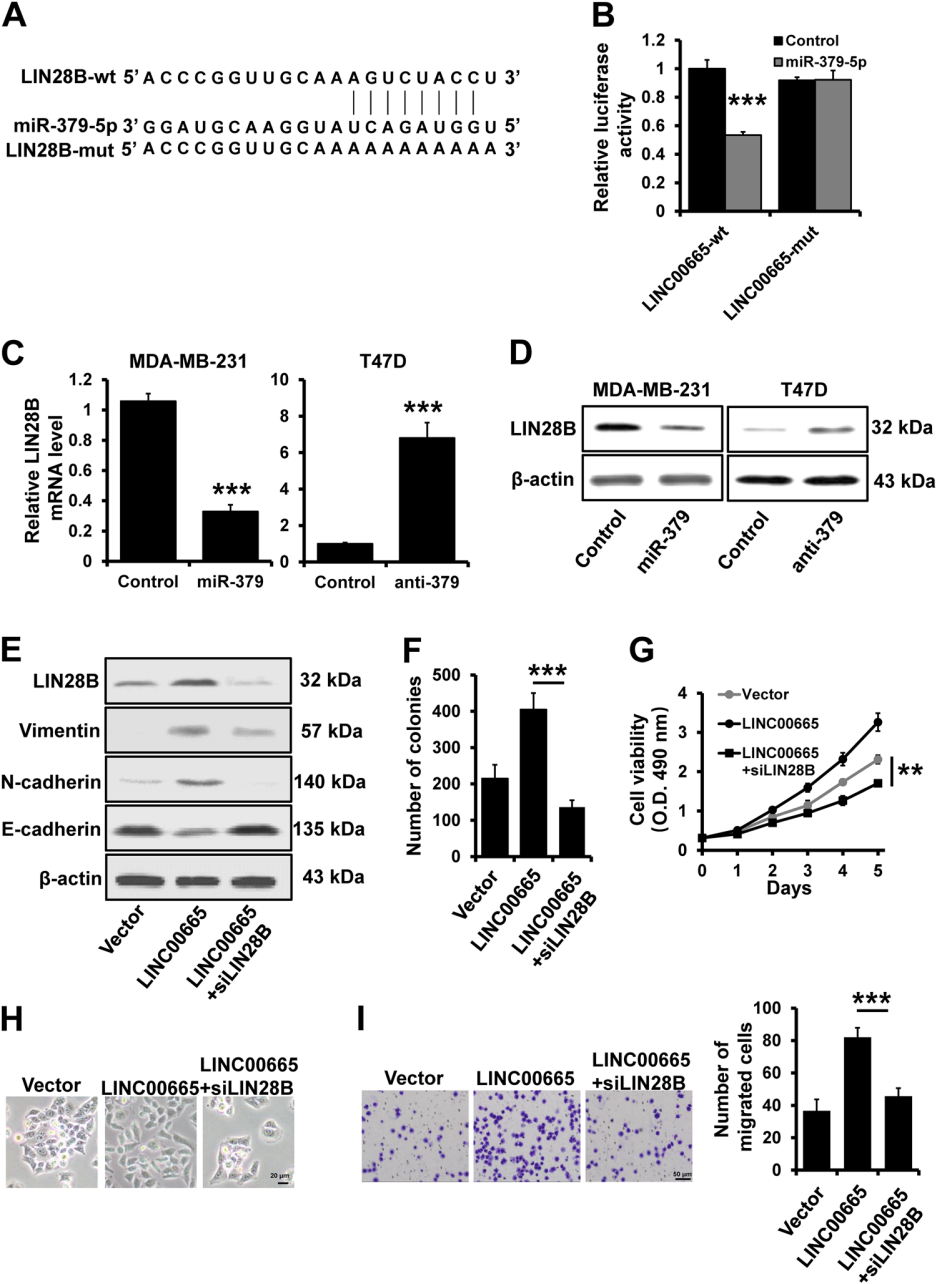
LINC00665 promotes breast cancer progression by regulating the miR-379-5p/axis. **a** The predicted binding site of miR-379-5p in the LIN28B 3ʹ-UTR. **b** A dual luciferase reporter assay was performed to validate LIN28B as a target of miR-379-5p. The mut sequence contains an 8-base mutation at the miR-379-5p target seed region. **c**, **d** The mRNA **c** and protein **d** expression levels of LIN28B in miR-379-5p-overexpressing MDA-MB-231 and miR-379-depleted T47D cells, as well as in the corresponding control cells, were determined by RT-qPCR and western blotting, respectively. **e** The expression levels of LIN28B and EMT markers in LINC00665-overexpressing T47D cells with or without transfection of siRNAs targeting LIN28B, as well as in the corresponding control cells, were determined by western blotting. **f**, **g** Growth inhibition was evaluated by colony formation **f** and MTT **g** assays in the cells described in **e**. **h** Images of the morphology of the cells described in **e**. **I** Transwell invasion assay of the cells described in **e**. ***P < 0.001, **P < 0.01.

LIN28B was identified as a putative miR-379-5p target by using starBase (Fig. 5a). To further confirm this regulatory relationship, the LIN28B 3′-UTR and a corresponding mutant construct containing a mutation in the putative miR-379-5p-binding site were cloned downstream of the luciferase ORF. Compared to control cells, miR-379-5p-transfected 293FT cells exhibited significantly decreased luciferase activity, with an inhibition rate of 40% (Fig. 5b; left). This effect was abolished in cells transfected with the mutated LIN28B 3ʹ-UTR, in which the binding site for miR-379-5p was mutated (Fig. 5b; right). Furthermore, we observed a decreased LIN28B expression in miR-379-5p-overexpressing MDA-MB-231 cells and an increased LIN28B expression in miR-379-5p-depleted T47D cells compared with that in control cells by RT-qPCR (Fig. 5c) and western blotting (Fig. 5d). Thus, these data indicate that LIN28B is a target of miR-379-5p.

### LINC00665 promotes breast cancer progression by regulating the miR-379-5p/LIN28B axis

We next investigated whether LINC00665 regulates breast cancer progression by regulating LIN28B expression. We transfected siRNAs targeting LIN28B into LINC00665-overexpressing T47D cells. The expression of LIN28B was increased in LINC00665-overexpressing cells but decreased after the transfection of siRNAs targeting LIN28B, as evidenced by western blotting (Fig. 5e). The MTT and colony formation assay results showed that depletion of LIN28B reversed the promotion of cell proliferation induced by LINC00665 in T47D cells (Fig. 5f, g). In addition, T47D cells transfected with pcDNA3.1-LINC00665 displayed a fibroblast-like morphology, whereas LIN28B-depleted cells retained their cobblestone-like morphology (Fig. 5h). Furthermore, LIN28B-depleted T47D cells exhibited significant upregulation of E-cadherin but dramatic downregulation of the mesenchymal markers Vimentin and N-cadherin, as evidenced by western blotting (Fig. 5e). Thus, when LIN28B expression was depleted, LINC00665 could no longer increase T47D cell invasion (Fig. 5i). LIN28B is responsible for the post-transcriptional downregulation of the let-7 in many types of cancers. We observed that let-7 is regulated by LINC00665/miR-379-5p/LIN28B axis (Fig. S4).

**Fig. 6:**
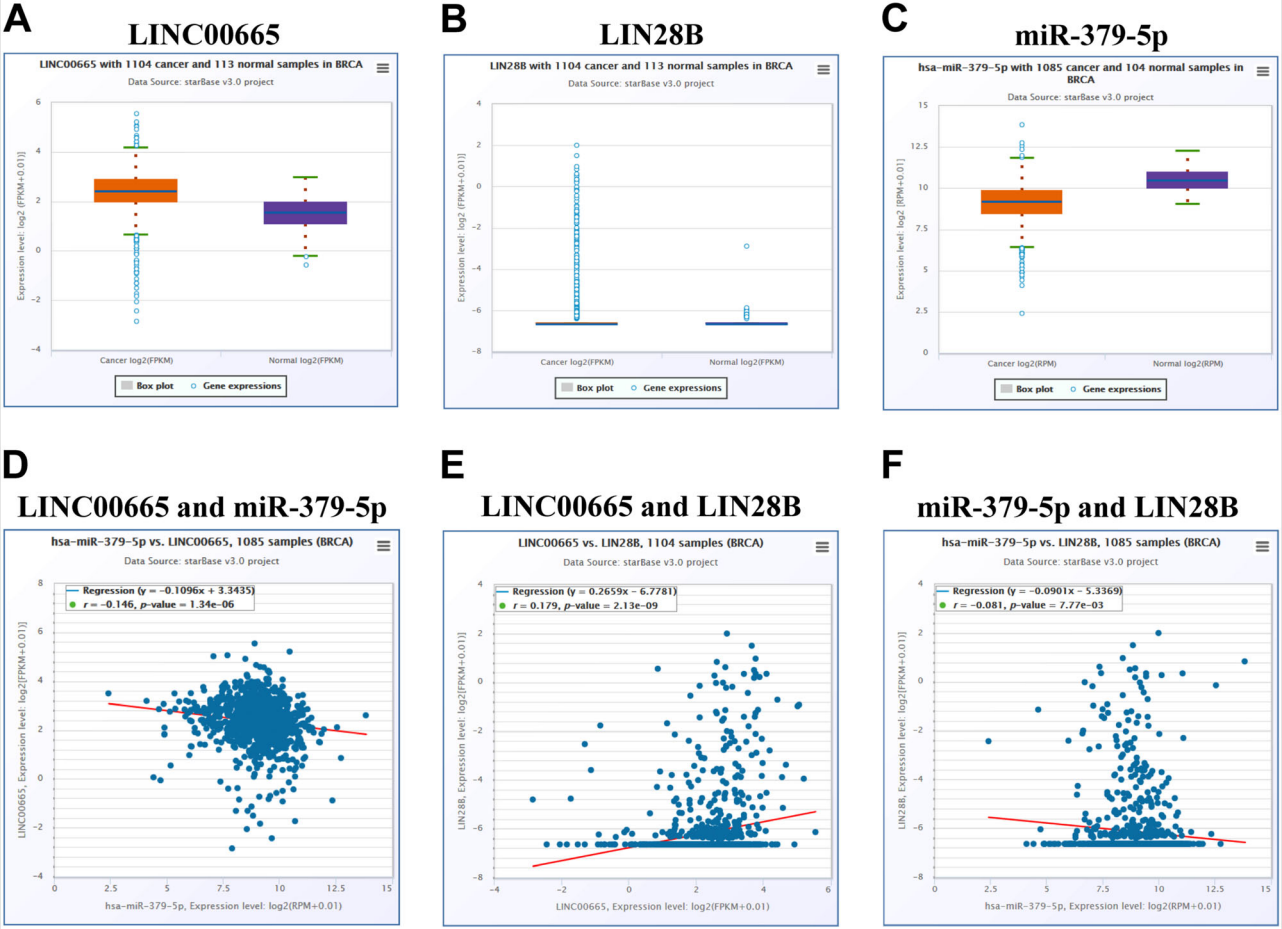
The clinical relevance of LINC00665, miR-379-5p, and LIN28B expression in breast cancer. **a**–**c** The expression of LINC00665 **a**, LIN28B **b**, and miR-379-5p **c** in breast cancer and normal breast tissues in the TCGA database. **d** and **e** The relationship between LINC00665, LIN28B, and miR-379-5p expression in breast cancer tissues in the TCGA database.

Finally, we determined the clinical relevance of LINC00665 expression in breast cancer. The expression of LINC00665, miR-379-5p, and LIN28B in breast cancer and normal breast tissues were obtained from the TCGA database and we found upregulation of LINC00665 and LIN28B expression and a downregulation of miR-379-5p expression in breast cancer tissues (Fig. 6a–c). More importantly, we observed a significant relationship among LINC00665, miR-379-5p, and LIN28B expression in the TCGA database (Fig. 6d–f). Taken together, our results indicate that LINC00665 promotes breast cancer progression through regulating the miR-379-5p/axis.

## Discussion

In the current study, we showed that LINC00665 functions as an oncogene in breast cancer. LINC00665 promotes breast cancer progression and induces an EMT-like phenotype. Furthermore, LIN28B is a target of miR-379-5p, and overexpression of LINC00665 promotes the expression of LIN28B by sponging miR-379-5p. Thus, our results revealed a LINC00665–miR-379-5p–LIN28B axis involved in the regulation of EMT during breast tumorigenesis and progression.

Dysfunction of lncRNAs is associated with multiple cellular processes during tumorigenesis and progression, such as apoptosis, proliferation, invasion, migration, angiogenesis, and metastasis^15^. Recently, LINC00665 has been identified to play an oncogenic role in lung cancer^13,14^ and liver cancer^12^. However, the role of LINC00665 in breast cancer is still unclear. Consistent with the results of previous studies in other cancers, our results provide evidence that LINC00665 is upregulated in breast cancer tissues, as evidenced by analysis of the TCGA database. Furthermore, we found that LINC00665 promotes breast cancer cell proliferation, migration, and invasion. EMT has been shown to play pivotal roles in cancer progression. During the EMT process, epithelial cells lose their cell polarity and cell adhesion, and acquire an invasive mesenchymal phenotype. Down-regulated expression of the epithelial marker E-cadherin and up-regulated expression of the mesenchymal markers vimentin and N-cadherin are often occurred during EMT^16^. In this study, LINC00665 reduced the expression of E-cadherin and induced the expression of N-cadherin and vimentin, whereas knockdown of endogenous LINC00665 produced the opposite effects in breast cancer cells, suggesting that LINC00665 is an inducer of EMT in breast cancer.

In addition to the regulatory effects of lncRNAs mediated through direct interaction with DNA, RNA, or proteins, the ceRNA hypothesis, which is supported by numerous studies, has been presented to describe a novel regulatory mechanism of RNAs^17,18^. LINC00665 acts as a sponge for miR-98 and promotes cancer progression in lung adenocarcinoma^13^. However, the mechanism of LINC00665 in breast cancer is unclear. Here, we provide evidence of a novel ceRNA-regulatory network in which LINC00665 functions as a sponge for miR-379-5p. Accumulating evidence indicates that numerous miRNAs frequently dysfunction in breast cancer and function as either tumor suppressors or oncogenes during breast tumorigenesis and progression; moreover, miRNAs are potential diagnostic and prognostic markers in patients with breast cancer^19,20^. For example, miR-379-5p has been identified as a tumor suppressor in several types of cancer, including bladder cancer^21^, liver cancer^22^, glioma^23^, gastric cancer^24^, osteosarcoma^25,26^, and breast cancer^27^. We present evidence that LINC00665 can promote breast cancer progression through inhibiting miR-379-5p expression. These results provide further evidence supporting the ceRNA regulatory network.

lncRNAs were reported to function as ceRNAs by acting as endogenous decoys for miRNAs, in turn affecting the binding of miRNAs to their targets^28^. LIN28B, an RNA-binding protein, functions as an oncogene and is a potential therapeutic target for cancer^29,30^. Upregulation of LIN28B promotes the conversion of epithelial cells to a more undifferentiated stage and maintains tumor cells in this stem-like stage^31,32^. In our study, we identified LIN28B as a direct target of miR-379-5p. Consistent with the function of LINC00665 as a sponge for miR-379-5p, we showed that the repression of LIN28B by miR-379-5p was partially rescued in the addition of LINC00665. Thus, the LINC00665–miR-379-5p–LIN28B axis may be a critical player during breast tumorigenesis and progression.

**Fig. 7:**
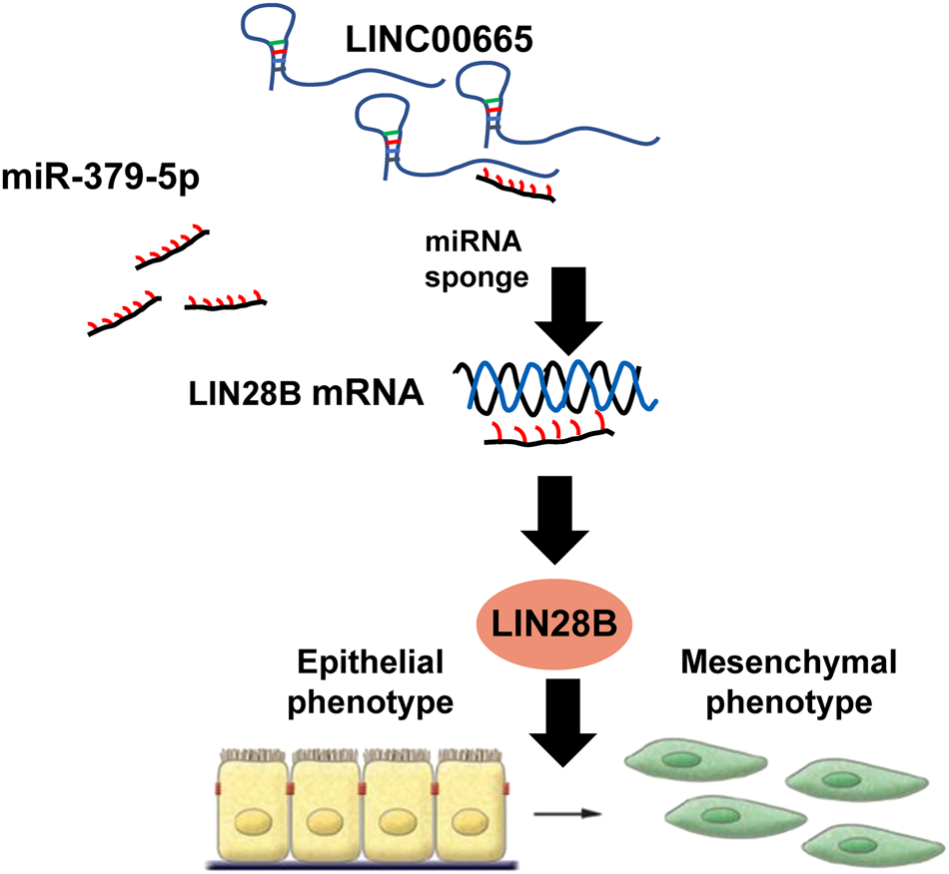
A model of the role of LINC00665 in breast cancer progression.

In conclusion, we demonstrated that LINC00665 functions as an oncogene in breast cancer. LINC00665 promotes breast cancer progression and induces an EMT-like phenotype. Furthermore, LINC00665 functions as a ceRNA to exert malignant characteristics in breast cancer through miR-379-5p–LIN28B axis. We propose a model that highlights the function of LINC00665 in regulating EMT during breast cancer progression (Fig. 7). Collectively, our results reveal that the LINC00665–miR-379-5p–LIN28B axis is a critical player in breast cancer progression and a promising target for breast cancer therapy.

## Materials and methods

### Cell culture

The normal human breast epithelial cell line MCF10A, human embryonic kidney cell line 293FT and six breast cancer cell lines (MCF7, BT474, BT549, MDA-MB-231, MDA-MB-468, and T47D) were obtained from the Cell Bank of the Chinese Academy of Sciences (Shanghai, China) and cultured as previously described^16^. All cell lines in the experiments were validated by STR DNA analysis and were negative for mycoplasma.

### Plasmids, miRNAs, and antibodies

The miR-379-5p inhibitor, miR-379-5p mimic, and the appropriate controls were obtained from RiboBio (Guangzhou, China). The 3ʹ-UTRs of LIN28B and LINC00665 containing the miR-379-5p-binding site were synthesized and subcloned into the psiCHECK2 vector (Promega, Madison, WI, USA) to generate the LIN28B-wt and LINC00665-wt constructs, respectively. QuikChange® Site-Directed Mutagenesis Kit (TransGene, Beijing, China) was used to generate the LIN28B 3ʹ-UTR mutant (LIN28B-mut) and LINC00665-mut reporter vectors according to the manufacturer’s instructions. Full-length LINC00665 was synthesized and cloned into the pcDNA3.1 expression vector. Anti-Vimentin antibody (D21H3), anti-N-cadherin (13A9), anti-E-cadherin (24E10), anti-LIN28B (D4H1), and anti-β-actin (8H10D10) were purchased from Cell Signaling Technology.

### Transfection and generation of the stable LINC00665-overexpressing cell line

Plasmids or miRNAs were transfected into different cell lines using TransFast transfection reagent (Promega) or FuGENE HD transfection reagent (Promega) according to the manufacturer’s instructions, respectively. To establish LINC00665-overexpressing cell line, 4 μg of either pcDNA3.1-LINC00665 or empty vector was transfected into T47D cells. Cells were then cultured in medium containing 1000 μg/mL G418 (Sigma-Aldrich, St. Louis, MO, USA) for 3 weeks. Monoclonal-resistant cell was obtained using a limiting dilution assay and maintained in medium supplemented with 500 μg/mL G418.

### Western blotting

Total protein was extracted by using RIPA buffer (50 mM Tris, 150 mM NaCl, 0.5% mM EDTA, 0.5% NP40) containing protease inhibitor cocktail tablet (Roche Molecular Biochemicals, Indianapolis, IN, USA) and centrifuged for 20 min at 12,000 rpm. 50 μg of total protein was loaded and separated on the 10% sodium dodecyl sulfate–polyacrylamide gradient gel. The proteins were then transferred onto PVDF membranes (Millipore, Bedford, MA, USA) and blocked with 5% non-fat milk at room temperature for 1 h. The membranes were then incubated at 4 °C overnight with primary antibodies against LIN28B, Vimentin, N-cadherin, E-cadherin, and β-actin were purchased from Cell Signaling Technology (Beverly, MA, USA). Then, the membranes were incubated with horseradish peroxidase-conjugated secondary antibody for 1 h at room temperature, and proteins were then detected using the ECL reagent (Millipore).

### Proliferation assays

MTT, EdU, and plate colony formation assays were used to evaluate the ability of cell proliferation, as previously described^33^.

### Fluorescence in situ hybridization

For FISH assay, the FISH Kit was purchased from RiboBio in line with the manufacturer’s recommendations. Fluorescence-conjugated LINC00665 probes were designed and synthesized by RiboBio. The cells were fixed in 4% formaldehyde and then washed with PBS. The fixed cells were permeabilized in PBS containing 0.5% Triton X-100 and prehybridized in prehybridization buffer. Next, cells were further incubated with 50 nM of the probe in hybridization buffer at 4 °C overnight. The next day, cells were washed with PBST and counterstained with 4′,6-diamidino-2-phenylindole (DAPI).

### RNA extraction and reverse transcription-quantitative PCR

Total RNA was extracted from cultured cells using a mirVana^TM^ PARIS^TM^ Kit (Life Technologies) according to the manufacturer’s instructions. TaqMan RT-qPCR was performed to detect the expression of mature miRNAs using a TaqMan miRNA Reverse Transcription Kit according to the manufacturer’s instructions (Life Technologies). qPCR was performed with GoTaq® qPCR Master Mix (Promega). The Ct values of each gene were averaged from triplicate reactions. The gene expression was determined by 2^−ΔCt^ method.

### Luciferase reporter assay

For the luciferase reporter assay, 293FT cells were co-transfected with miR-379-5p mimic or mimic control and 1000 ng of LIN28B-wt/mut or LINC00665-wt/mut. Cells were seeded in 24-well plates and collected 48 h after transfection. The luciferase activity was determined by a Dual-Luciferase Reporter Assay System (Promega) according to the manufacturer's recommendations, as previously described^34^. All transfections were performed in triplicate.

### Transwell and wound healing assays

The cell invasive ability was evaluated in Transwell chambers coated with Matrigel (BD Biosciences, San Diego, CA, USA) as previously described^16^. For the wound-healing assay, cells were cultured in six-well plates to 70–80% confluency and were wounded with a 200-μl sterile pipette tip. After washing with PBS, the cells were cultured in serum-free medium. Images were acquired at each time point.

### Xenograft model

Female SCID mice (5 weeks age, five mice per group) were subcutaneously injected with tumor cells (2 × 10^6^ cells) containing 100 μg of Matrigel (BD Biosciences) into the mammary fat pads. Tumors formed by tumor cells were measured with a caliper-like instrument and tumor volumes were calculated according to the following formula: Volume (mm^3^) = width^2^ (mm^2^) × length (mm)/2. In consideration of animal welfare, mice were sacrificed at 5 weeks, and the final volumes and weights of tumor tissues were determined. All animal studies were approved by the Animal Ethics Committee of Tianjin Medical University Cancer Institute and Hospital and were performed according to both the guidelines for the welfare and use of animals in cancer research and national law.

### Immunohistochemistry

Mouse tumors were fixed with 4% paraformaldehyde at 4 °C overnight and were then embedded in paraffin. Serial sections (2 μm thick) were incubated with primary antibodies (anti-Vimentin and anti-E-cadherin) for 2 h at room temperature and subsequently incubated with anti-mouse HRP-conjugated secondary antibody for 1 h at room temperature. After three rinses with PBS, slides were stained with diaminobenzidine followed by counterstaining with hematoxylin.

### Statistical analysis

Data are presented as the means ± standard deviations from at least three independent experiments. All calculations were performed with IBM SPSS Statistics for Windows (IBM Corp., Armonk, NY, USA). Differences between groups were analyzed by using the Student’s *t*-test and *P*-values < 0.05 were considered as statistically significant.

## Supplementary information

Supplemental material
