# Supplementary material for: LINC00665 promotes breast cancer progression through regulation of the miR-379-5p/LIN28B axis
Source: Cell Death Dis. 2020 Jan 6;11(1):16. doi: 10.1038/s41419-019-2213-x (PMC6944690; doi:10.1038/s41419-019-2213-x)
Supplement: Supplementary file 1 — Supplemental material [file 41419_2019_2213_MOESM1_ESM.docx]

**
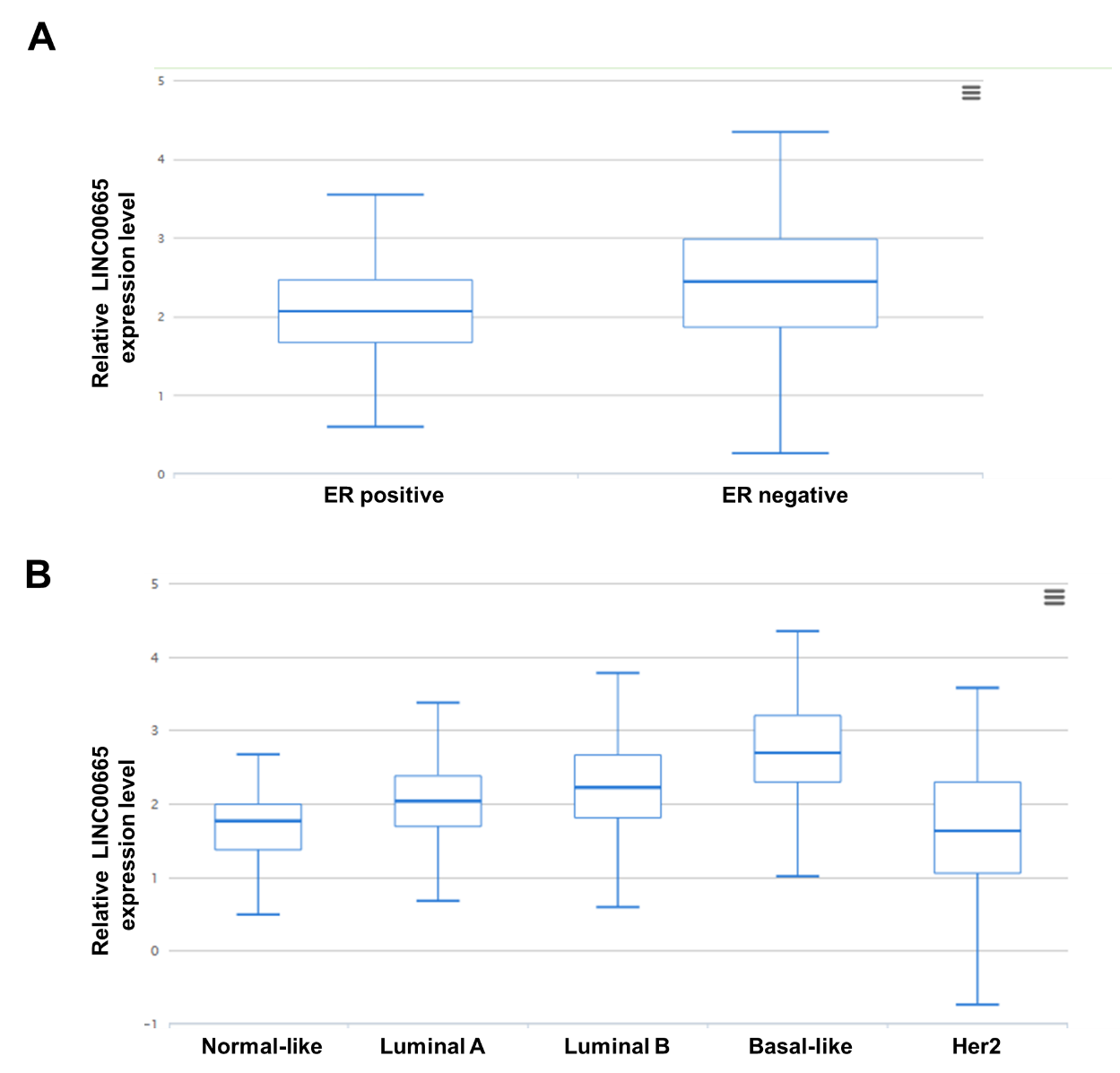
**

**Figure S1. The expression of LINC00665 in breast cancer specimens from TCGA database. A,** The expression of LINC00665 breast cancer specimens with different ER status. **B,** The expression of LINC00665 breast cancer specimens with different molecular subtypes.


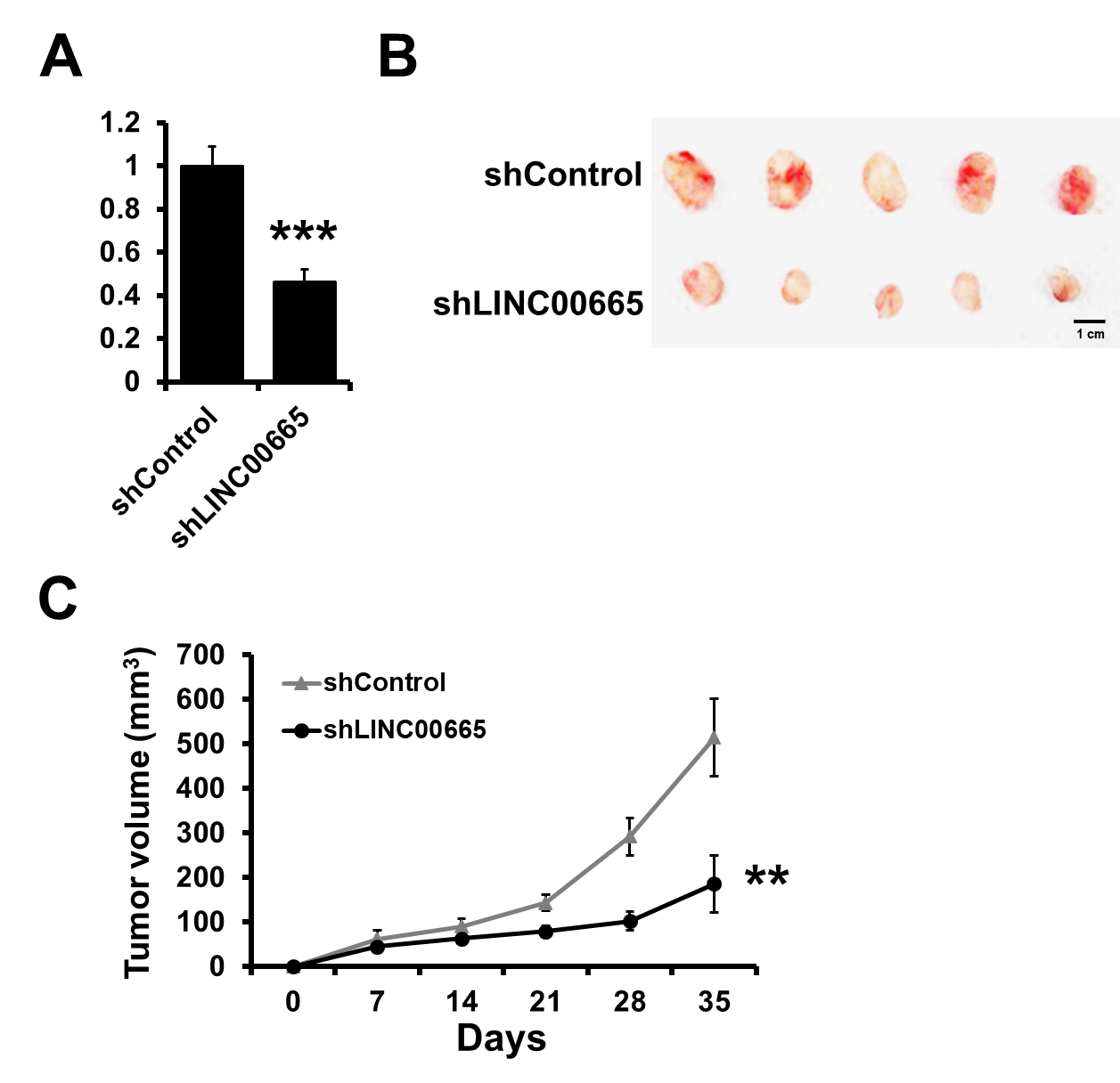


**Figure S2. Depletion of LINC00665 inhibits tumor growth in MDA-MB-231 cells.** **A,** LINC00665 expression in MDA-MB-231 cells transfected with the shControl or shRNA targeting LINC00665 was determined by RT-qPCR. **B,** Representative photographs of the tumors formed by 231-shLINC00665 or shControl cells at the time of harvest. **C,** Volume of tumors in xenograft mice injected with 231-shLINC00665 or shControl cells at the indicated times. ****P* < 0.001, ***P* < 0.01.


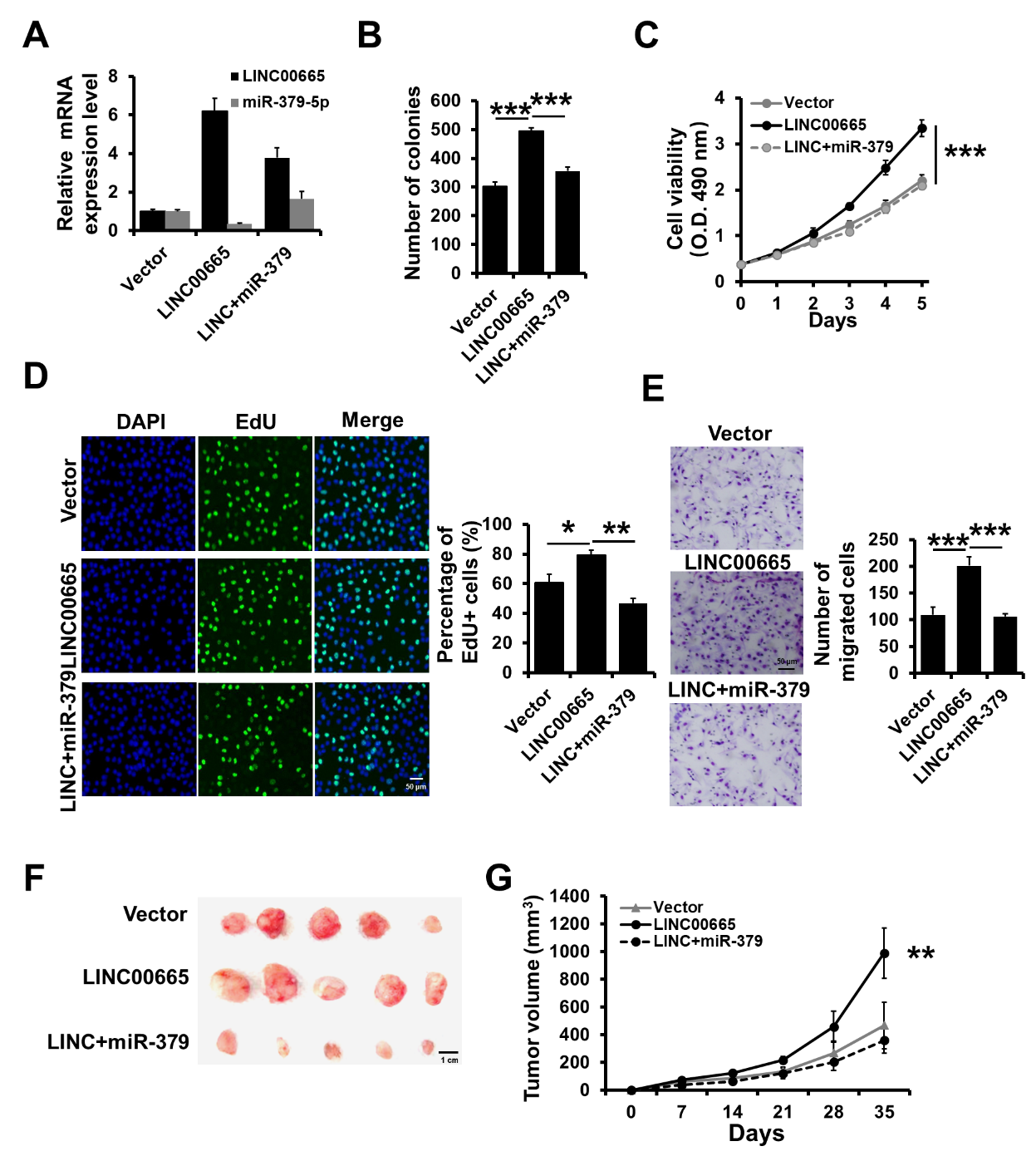


**Figure S3.** **LINC00665 promotes breast cancer progression by regulation of miR-379-5p expression. A,** LINC00665 and miR-379-5p expression in MDA-MB-231 cells transfected with the LINC00665 (and miR-379-5p) expression vector or empty vector was determined by RT-qPCR. **B-D,** Growth inhibition was evaluated by colony formation (B), MTT (C), and EdU (D) assays in the cells described in (A). **E,** Transwell invasion assay of the cells described in (A). **F,** Representative photographs of the tumors formed by 231-LINC00665, 231-LINC00665/miR-379-5p or control cells at the time of harvest. **G,** Volume of tumors in xenograft mice injected with 231-LINC00665, 231-LINC00665/miR-379-5p or control cells at the indicated times. ****P* < 0.001, ***P* < 0.01, **P* < 0.05.


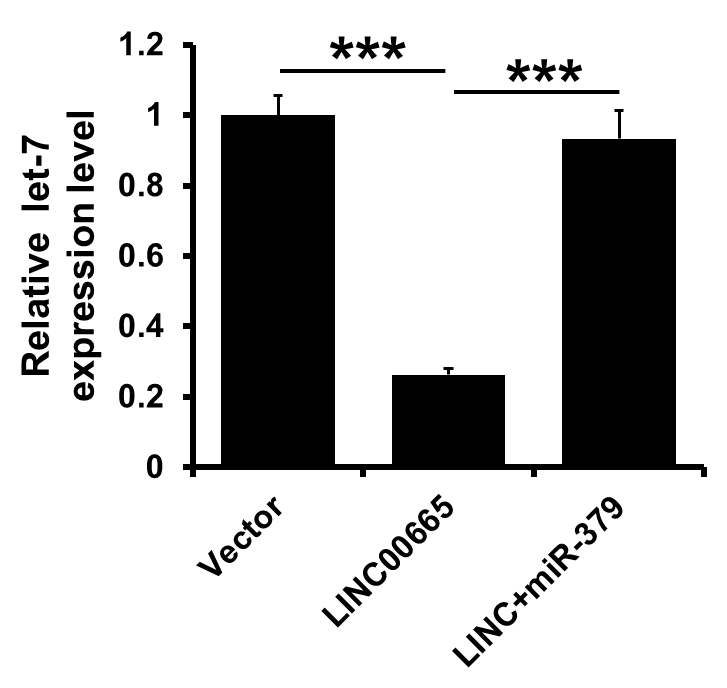


**Figure S4.** **The expression of let-7 in indicated cells.** ****P* < 0.001
